# *N*-Benzyl-*N*,4-dimethyl­benzene­sulfonamide

**DOI:** 10.1107/S1600536810044156

**Published:** 2010-11-06

**Authors:** Islam Ullah Khan, Mehmet Akkurt, Shahzad Sharif, Waqar Ahmad

**Affiliations:** a Department of Chemistry, Government College University, Lahore 54000, Pakistan; b Department of Physics, Faculty of Sciences, Erciyes University, 38039 Kayseri, Turkey

## Abstract

The mol­ecule of the title compound, C_15_H_17_NO_2_S, has a C—S—N—C torsion angle of 71.4 (2)°, and the dihedral angle between the benzene rings is 82.83 (16)°. In the crystal, mol­ecules are linked into chains along the *b* axis *via* C—H⋯O hydrogen bonds. A C—H⋯π inter­action is also present in the crystal structure.

## Related literature

For the pharmacological activities of sulfonamides, see: Maren (1976 ▶); Boyd (1988 ▶). For our previous studies on derivatives of sulfonamide, see: Khan, Ahmad, Arshad *et al.* (2010 ▶); Khan, Ahmad, Sharif *et al.* (2010 ▶).
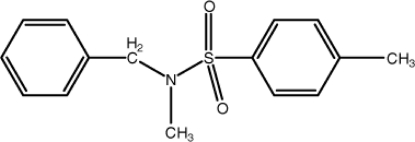


## Experimental

### 

#### Crystal data

C_15_H_17_NO_2_S
*M* *_r_* = 275.37
Monoclinic, 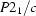

*a* = 15.0386 (16) Å
*b* = 8.2632 (7) Å
*c* = 12.0758 (12) Å
β = 105.902 (4)°
*V* = 1443.2 (2) Å^3^
*Z* = 4
Mo *K*α radiation
μ = 0.22 mm^−1^
*T* = 296 K
0.28 × 0.17 × 0.09 mm

#### Data collection

Bruker APEXII CCD diffractometer
13504 measured reflections
3569 independent reflections
1580 reflections with *I* > 2σ(*I*)
*R*_int_ = 0.042

#### Refinement

*R*[*F* ^2^ > 2σ(*F* ^2^)] = 0.052
*wR*(*F* ^2^) = 0.155
*S* = 1.01
3569 reflections
174 parameters
H-atom parameters constrained
Δρ_max_ = 0.18 e Å^−3^
Δρ_min_ = −0.24 e Å^−3^

### 

Data collection: *APEX2* (Bruker, 2007 ▶); cell refinement: *SAINT* (Bruker, 2007 ▶); data reduction: *SAINT*; program(s) used to solve structure: *SHELXS97* (Sheldrick, 2008 ▶); program(s) used to refine structure: *SHELXL97* (Sheldrick, 2008 ▶); molecular graphics: *ORTEP-3 for Windows* (Farrugia, 1997 ▶); software used to prepare material for publication: *WinGX* (Farrugia, 1999 ▶) and *PLATON* (Spek, 2009 ▶).

## Supplementary Material

Crystal structure: contains datablocks global, I. DOI: 10.1107/S1600536810044156/xu5070sup1.cif

Structure factors: contains datablocks I. DOI: 10.1107/S1600536810044156/xu5070Isup2.hkl

Additional supplementary materials:  crystallographic information; 3D view; checkCIF report

## Figures and Tables

**Table 1:** Hydrogen-bond geometry (Å, °) *Cg*2 is the centroid of the C10–C15 phenyl ring.

*D*—H⋯*A*	*D*—H	H⋯*A*	*D*⋯*A*	*D*—H⋯*A*
C6—H6⋯O1^i^	0.93	2.53	3.414 (3)	158
C9—H9*A*⋯*Cg*2^ii^	0.97	2.99	3.721 (3)	133

Symmetry codes: (i) 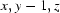; (ii) 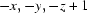.

**Fig. 1.**
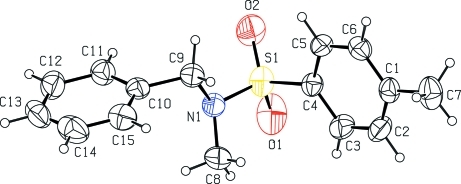
The title molecule showing the atomic numbering scheme and 30% probability displacement ellipsoids.

**Fig. 2.**
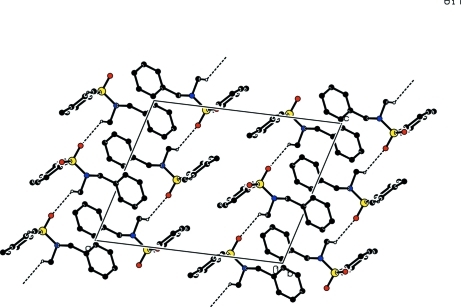
The packing and hydrogen bonding of the title compound in the unit cell. Hydrogen bonds are shown as dashed lines. For the sake of clarity, the H atoms not involved in the motif have been omitted.

## supplementary crystallographic information

### Comment

Sulfonamides have extensively been reported for their wide variety of pharmacological activities such as antibacterial (Maren, 1976) and diuretic (Boyd, 1988). The present structure is continuous to our previous reported derivative of sulfonamide (Khan, Ahmad, Arshad *et al.**et al.*, 2010; Khan, Ahmad, Sharif *et al.*, 2010).

In the title molecule (I), (Fig. 1), the molecule has a C4—S1—N1—C9 torsion angle of 71.4 (2)°. The dihedral angle between the sulfonyl benzene ring (C1–C6) and the phenyl ring (C10–C15) is 82.83 (16)°. In the structure, molecules are linked into chains along the *b* axis *via* C—H···O hydrogen bonding (Table 1, Fig. 2). In the structure, there is a C—H···π interaction (Table 1).

### Experimental

A mixture of *N*-benzyl-4-methylbenzenesulfonamide (0.5 g, 2.02 mmol) and sodium hydride (0.2 g, 8.333 mmol) in N,N-dimethylformamide (10 ml) was stirred at room temperature for 30 min followed by the addition of methyl iodide (0.25 ml, 2.02 mmol). After the consumption of reactants (as monitored by TLC), the contents were poured over crushed ice. The precipitated product was isolated, washed, dried and recrystallized from chloroform solution to yield colourless blocks of title compound.

### Refinement

All H atoms were positioned geometrically with C—H = 0.93–0.97 Å and treated as riding on their parent atoms, with *U*_iso_(H) = 1.2 or 1.5*U*_eq_(C).

### Crystal data

**Table:**

C_15_H_17_NO_2_S	*F*(000) = 584
*M**_r_* = 275.37	*D*_x_ = 1.267 Mg m^−^^3^
Monoclinic, *P*2_1_/*c*	Mo *K*α radiation, λ = 0.71073 Å
Hall symbol: -P 2ybc	Cell parameters from 1987 reflections
*a* = 15.0386 (16) Å	θ = 2.8–19.5°
*b* = 8.2632 (7) Å	µ = 0.22 mm^−^^1^
*c* = 12.0758 (12) Å	*T* = 296 K
β = 105.902 (4)°	Block, colourless
*V* = 1443.2 (2) Å^3^	0.28 × 0.17 × 0.09 mm
*Z* = 4	

### Data collection

**Table:**

Bruker APEXII CCD diffractometer	1580 reflections with *I* > 2σ(*I*)
Radiation source: sealed tube	*R*_int_ = 0.042
graphite	θ_max_ = 28.3°, θ_min_ = 1.4°
φ and ω scans	*h* = −19→20
13504 measured reflections	*k* = −9→11
3569 independent reflections	*l* = −14→16

### Refinement

**Table:**

Refinement on *F*^2^	Primary atom site location: structure-invariant direct methods
Least-squares matrix: full	Secondary atom site location: difference Fourier map
*R*[*F*^2^ > 2σ(*F*^2^)] = 0.052	Hydrogen site location: inferred from neighbouring sites
*wR*(*F*^2^) = 0.155	H-atom parameters constrained
*S* = 1.01	*w* = 1/[σ^2^(*F*_o_^2^) + (0.0492*P*)^2^ + 0.4014*P*] where *P* = (*F*_o_^2^ + 2*F*_c_^2^)/3
3569 reflections	(Δ/σ)_max_ < 0.001
174 parameters	Δρ_max_ = 0.18 e Å^−^^3^
0 restraints	Δρ_min_ = −0.24 e Å^−^^3^

### Special details

**Table:**

Geometry. Bond distances, angles *etc*. have been calculated using the rounded fractional coordinates. All su's are estimated from the variances of the (full) variance-covariance matrix. The cell e.s.d.'s are taken into account in the estimation of distances, angles and torsion angles
Refinement. Refinement on *F*^2^ for ALL reflections except those flagged by the user for potential systematic errors. Weighted *R*-factors *wR* and all goodnesses of fit *S* are based on *F*^2^, conventional *R*-factors *R* are based on *F*, with *F* set to zero for negative *F*^2^. The observed criterion of *F*^2^ > σ(*F*^2^) is used only for calculating -*R*-factor-obs *etc*. and is not relevant to the choice of reflections for refinement. *R*-factors based on *F*^2^ are statistically about twice as large as those based on *F*, and *R*-factors based on ALL data will be even larger.

### Fractional atomic coordinates and isotropic or equivalent isotropic displacement parameters (Å^2^)

**Table:**

	*x*	*y*	*z*	*U*_iso_*/*U*_eq_	
S1	0.28540 (5)	0.19774 (9)	0.52072 (8)	0.0817 (3)	
O1	0.33542 (16)	0.3390 (2)	0.5047 (3)	0.1286 (12)	
O2	0.27578 (16)	0.1610 (3)	0.63199 (19)	0.1120 (10)	
N1	0.18117 (15)	0.2182 (2)	0.4348 (2)	0.0696 (9)	
C1	0.40480 (17)	−0.2407 (4)	0.3876 (2)	0.0683 (10)	
C2	0.41527 (19)	−0.0886 (4)	0.3473 (3)	0.0836 (12)	
C3	0.38139 (19)	0.0469 (4)	0.3880 (3)	0.0799 (11)	
C4	0.33502 (16)	0.0309 (3)	0.4717 (2)	0.0603 (9)	
C5	0.32447 (17)	−0.1215 (3)	0.5129 (2)	0.0647 (10)	
C6	0.35966 (18)	−0.2545 (3)	0.4714 (2)	0.0685 (10)	
C7	0.4415 (2)	−0.3883 (4)	0.3416 (3)	0.1029 (16)	
C8	0.1774 (2)	0.2578 (4)	0.3159 (3)	0.1013 (14)	
C9	0.11230 (18)	0.0967 (3)	0.4439 (3)	0.0719 (10)	
C10	0.01606 (19)	0.1670 (3)	0.4037 (2)	0.0644 (10)	
C11	−0.0138 (2)	0.2751 (4)	0.4709 (3)	0.0804 (12)	
C12	−0.1020 (3)	0.3421 (4)	0.4332 (4)	0.0991 (16)	
C13	−0.1597 (2)	0.2996 (5)	0.3276 (5)	0.1090 (18)	
C14	−0.1292 (3)	0.1911 (5)	0.2621 (4)	0.1128 (17)	
C15	−0.0424 (2)	0.1256 (4)	0.2990 (3)	0.0876 (12)	
H2	0.44620	−0.07680	0.29080	0.1000*	
H3	0.38960	0.14860	0.35930	0.0960*	
H5	0.29330	−0.13430	0.56900	0.0780*	
H6	0.35270	−0.35620	0.50080	0.0820*	
H7A	0.47420	−0.45520	0.40460	0.1540*	
H7B	0.48270	−0.35560	0.29750	0.1540*	
H7C	0.39090	−0.44830	0.29330	0.1540*	
H8A	0.19200	0.16320	0.27810	0.1520*	
H8B	0.22130	0.34170	0.31490	0.1520*	
H8C	0.11630	0.29440	0.27650	0.1520*	
H9A	0.12430	0.06140	0.52320	0.0860*	
H9B	0.11700	0.00330	0.39720	0.0860*	
H11	0.02490	0.30430	0.54230	0.0970*	
H12	−0.12190	0.41580	0.47950	0.1190*	
H13	−0.21850	0.34440	0.30170	0.1310*	
H14	−0.16780	0.16060	0.19100	0.1350*	
H15	−0.02290	0.05200	0.25230	0.1050*	

### Atomic displacement parameters (Å^2^)

**Table:**

	*U*^11^	*U*^22^	*U*^33^	*U*^12^	*U*^13^	*U*^23^
S1	0.0745 (6)	0.0657 (5)	0.1035 (7)	−0.0062 (4)	0.0218 (4)	−0.0202 (4)
O1	0.1010 (18)	0.0631 (13)	0.222 (3)	−0.0296 (12)	0.0449 (18)	−0.0325 (16)
O2	0.1209 (19)	0.139 (2)	0.0748 (15)	0.0202 (15)	0.0246 (13)	−0.0337 (14)
N1	0.0700 (15)	0.0538 (13)	0.0903 (17)	0.0006 (11)	0.0308 (13)	0.0105 (12)
C1	0.0508 (16)	0.081 (2)	0.0685 (18)	0.0036 (13)	0.0088 (14)	−0.0079 (15)
C2	0.072 (2)	0.102 (2)	0.089 (2)	−0.0027 (17)	0.0428 (17)	0.0009 (19)
C3	0.075 (2)	0.0688 (19)	0.105 (2)	−0.0117 (15)	0.0399 (18)	0.0143 (17)
C4	0.0522 (15)	0.0594 (16)	0.0693 (17)	−0.0067 (11)	0.0168 (13)	−0.0028 (13)
C5	0.0607 (16)	0.0711 (18)	0.0654 (17)	−0.0030 (13)	0.0223 (13)	0.0058 (14)
C6	0.0670 (17)	0.0595 (16)	0.0749 (19)	0.0011 (13)	0.0127 (15)	0.0067 (14)
C7	0.088 (2)	0.109 (3)	0.106 (3)	0.0236 (19)	0.017 (2)	−0.027 (2)
C8	0.100 (2)	0.098 (2)	0.115 (3)	0.0158 (18)	0.045 (2)	0.051 (2)
C9	0.0745 (19)	0.0558 (15)	0.089 (2)	−0.0018 (13)	0.0286 (16)	0.0104 (14)
C10	0.0669 (18)	0.0530 (15)	0.0741 (19)	−0.0062 (13)	0.0209 (15)	0.0130 (14)
C11	0.073 (2)	0.081 (2)	0.086 (2)	−0.0004 (16)	0.0199 (17)	0.0049 (18)
C12	0.086 (3)	0.086 (2)	0.138 (3)	0.0105 (19)	0.052 (3)	0.022 (2)
C13	0.063 (2)	0.104 (3)	0.153 (4)	0.003 (2)	0.018 (3)	0.065 (3)
C14	0.089 (3)	0.122 (3)	0.110 (3)	−0.017 (2)	−0.002 (2)	0.031 (3)
C15	0.086 (2)	0.085 (2)	0.087 (2)	−0.0165 (18)	0.0154 (19)	0.0012 (18)

### Geometric parameters (Å, °)

**Table:**

S1—O1	1.430 (2)	C14—C15	1.370 (6)
S1—O2	1.423 (2)	C2—H2	0.9300
S1—N1	1.634 (2)	C3—H3	0.9300
S1—C4	1.746 (3)	C5—H5	0.9300
N1—C8	1.459 (4)	C6—H6	0.9300
N1—C9	1.468 (3)	C7—H7A	0.9600
C1—C2	1.372 (5)	C7—H7B	0.9600
C1—C6	1.369 (4)	C7—H7C	0.9600
C1—C7	1.506 (5)	C8—H8A	0.9600
C2—C3	1.376 (5)	C8—H8B	0.9600
C3—C4	1.383 (4)	C8—H8C	0.9600
C4—C5	1.379 (3)	C9—H9A	0.9700
C5—C6	1.373 (4)	C9—H9B	0.9700
C9—C10	1.511 (4)	C11—H11	0.9300
C10—C11	1.363 (4)	C12—H12	0.9300
C10—C15	1.371 (4)	C13—H13	0.9300
C11—C12	1.394 (6)	C14—H14	0.9300
C12—C13	1.377 (7)	C15—H15	0.9300
C13—C14	1.356 (6)		
			
O1···C6^i^	3.414 (3)	H2···H7B^x^	2.5000
O1···C7^ii^	3.384 (4)	H3···O1	2.6500
O2···C8^iii^	3.061 (4)	H5···O2	2.5900
O1···H6^i^	2.5300	H5···C13^ix^	2.9700
O1···H8B	2.4600	H6···O1^v^	2.5300
O1···H3	2.6500	H6···H7A	2.5500
O2···H5	2.5900	H7A···H6	2.5500
O2···H9A	2.4400	H7A···H7A^xi^	2.3400
O2···H7C^iv^	2.8300	H7B···H2	2.3600
O2···H8A^iii^	2.8300	H7B···H2^viii^	2.5000
O2···H8B^iii^	2.5600	H7C···O2^xii^	2.8300
C3···C8	3.427 (5)	H8A···C3	2.9500
C5···C9	3.559 (4)	H8A···C4	2.9200
C6···O1^v^	3.414 (3)	H8A···H9B	2.4400
C7···O1^ii^	3.384 (4)	H8A···O2^vi^	2.8300
C8···C15	3.434 (5)	H8B···O1	2.4600
C8···C3	3.427 (5)	H8B···O2^vi^	2.5600
C8···O2^vi^	3.061 (4)	H8C···C10	2.6500
C9···C5	3.559 (4)	H8C···C15	2.8400
C15···C8	3.434 (5)	H8C···C15^xiii^	3.0000
C2···H13^vii^	3.0600	H8C···H15^xiii^	2.5200
C3···H8A	2.9500	H9A···O2	2.4400
C4···H8A	2.9200	H9A···H11	2.5500
C6···H14^vii^	3.0900	H9B···H8A	2.4400
C7···H2^viii^	3.0500	H9B···H15	2.3700
C10···H8C	2.6500	H11···H9A	2.5500
C13···H5^ix^	2.9700	H13···C2^xiii^	3.0600
C15···H8C	2.8400	H14···C6^xiii^	3.0900
C15···H8C^vii^	3.0000	H15···H9B	2.3700
H2···H7B	2.3600	H15···H8C^vii^	2.5200
H2···C7^x^	3.0500		
			
O1—S1—O2	119.68 (18)	C4—C5—H5	120.00
O1—S1—N1	106.15 (14)	C6—C5—H5	120.00
O1—S1—C4	108.00 (14)	C1—C6—H6	119.00
O2—S1—N1	107.07 (14)	C5—C6—H6	119.00
O2—S1—C4	108.37 (13)	C1—C7—H7A	110.00
N1—S1—C4	106.92 (11)	C1—C7—H7B	110.00
S1—N1—C8	114.8 (2)	C1—C7—H7C	109.00
S1—N1—C9	117.09 (18)	H7A—C7—H7B	109.00
C8—N1—C9	112.9 (2)	H7A—C7—H7C	109.00
C2—C1—C6	117.8 (3)	H7B—C7—H7C	109.00
C2—C1—C7	121.4 (3)	N1—C8—H8A	109.00
C6—C1—C7	120.7 (3)	N1—C8—H8B	109.00
C1—C2—C3	121.9 (3)	N1—C8—H8C	109.00
C2—C3—C4	119.6 (3)	H8A—C8—H8B	110.00
S1—C4—C3	121.4 (2)	H8A—C8—H8C	109.00
S1—C4—C5	119.77 (19)	H8B—C8—H8C	110.00
C3—C4—C5	118.8 (2)	N1—C9—H9A	110.00
C4—C5—C6	120.4 (2)	N1—C9—H9B	110.00
C1—C6—C5	121.5 (2)	C10—C9—H9A	110.00
N1—C9—C10	110.3 (2)	C10—C9—H9B	110.00
C9—C10—C11	120.2 (3)	H9A—C9—H9B	108.00
C9—C10—C15	121.1 (3)	C10—C11—H11	120.00
C11—C10—C15	118.7 (3)	C12—C11—H11	120.00
C10—C11—C12	120.3 (3)	C11—C12—H12	120.00
C11—C12—C13	120.2 (4)	C13—C12—H12	120.00
C12—C13—C14	118.7 (4)	C12—C13—H13	121.00
C13—C14—C15	121.1 (4)	C14—C13—H13	121.00
C10—C15—C14	121.0 (3)	C13—C14—H14	120.00
C1—C2—H2	119.00	C15—C14—H14	119.00
C3—C2—H2	119.00	C10—C15—H15	120.00
C2—C3—H3	120.00	C14—C15—H15	119.00
C4—C3—H3	120.00		
			
O1—S1—N1—C8	50.6 (2)	C7—C1—C2—C3	179.5 (3)
O2—S1—N1—C8	179.5 (2)	C1—C2—C3—C4	−0.2 (5)
C4—S1—N1—C8	−64.5 (2)	C2—C3—C4—C5	0.4 (4)
O1—S1—N1—C9	−173.5 (2)	C2—C3—C4—S1	−176.6 (2)
O2—S1—N1—C9	−44.5 (2)	S1—C4—C5—C6	177.2 (2)
C4—S1—N1—C9	71.4 (2)	C3—C4—C5—C6	0.1 (4)
O1—S1—C4—C3	−26.5 (3)	C4—C5—C6—C1	−0.9 (4)
O2—S1—C4—C3	−157.5 (2)	N1—C9—C10—C11	−74.0 (3)
N1—S1—C4—C3	87.4 (2)	N1—C9—C10—C15	105.2 (3)
O1—S1—C4—C5	156.6 (2)	C9—C10—C11—C12	179.0 (3)
O2—S1—C4—C5	25.5 (2)	C15—C10—C11—C12	−0.2 (5)
N1—S1—C4—C5	−89.6 (2)	C9—C10—C15—C14	−179.2 (3)
C8—N1—C9—C10	−68.8 (3)	C11—C10—C15—C14	0.0 (5)
S1—N1—C9—C10	154.48 (19)	C10—C11—C12—C13	0.0 (6)
C6—C1—C2—C3	−0.5 (4)	C11—C12—C13—C14	0.5 (6)
C7—C1—C6—C5	−178.9 (3)	C12—C13—C14—C15	−0.7 (6)
C2—C1—C6—C5	1.1 (4)	C13—C14—C15—C10	0.4 (6)

Symmetry codes: (i) *x*, *y*+1, *z*; (ii) −*x*+1, −*y*, −*z*+1; (iii) *x*, −*y*+1/2, *z*+1/2; (iv) *x*, −*y*−1/2, *z*+1/2; (v) *x*, *y*−1, *z*; (vi) *x*, −*y*+1/2, *z*−1/2; (vii) −*x*, *y*−1/2, −*z*+1/2; (viii) −*x*+1, *y*−1/2, −*z*+1/2; (ix) −*x*, −*y*, −*z*+1; (x) −*x*+1, *y*+1/2, −*z*+1/2; (xi) −*x*+1, −*y*−1, −*z*+1; (xii) *x*, −*y*−1/2, *z*−1/2; (xiii) −*x*, *y*+1/2, −*z*+1/2.

### Hydrogen-bond geometry (Å, °)

**Table:**

Cg2 is the centroid of the C10–C15 phenyl ring.

**Table:**

*D*—H···*A*	*D*—H	H···*A*	*D*···*A*	*D*—H···*A*
C5—H5···O2	0.93	2.59	2.938 (3)	103
C6—H6···O1^v^	0.93	2.53	3.414 (3)	158
C8—H8B···O1	0.96	2.46	2.886 (5)	107
C8—H8B···O2^vi^	0.96	2.56	3.061 (4)	113
C9—H9A···O2	0.97	2.44	2.903 (4)	109
C9—H9A···Cg2^ix^	0.97	2.99	3.721 (3)	133

Symmetry codes: (v) *x*, *y*−1, *z*; (vi) *x*, −*y*+1/2, *z*−1/2; (ix) −*x*, −*y*, −*z*+1.
